# Illicit Stimulant Use Is Associated with Abnormal Substantia Nigra Morphology in Humans

**DOI:** 10.1371/journal.pone.0056438

**Published:** 2013-02-13

**Authors:** Gabrielle Todd, Carolyn Noyes, Stanley C. Flavel, Chris B. Della Vedova, Peter Spyropoulos, Barry Chatterton, Daniela Berg, Jason M. White

**Affiliations:** 1 School of Pharmacy and Medical Sciences, University of South Australia, Adelaide, South Australia, Australia; 2 Sansom Institute, University of South Australia, Adelaide, South Australia, Australia; 3 Department of Nuclear Medicine, PET & Bone Densitometry, and Radiology, Royal Adelaide Hospital, Adelaide, South Australia, Australia; 4 Department of Neurodegeneration, Hertie Institute for Clinical Brain Research and German Center of Neurodegenerative Diseases, Tübingen, Germany; College of Tropical Agriculture and Human Resources, University of Hawaii, United States of America

## Abstract

Use of illicit stimulants such as methamphetamine, cocaine, and ecstasy is an increasing health problem. Chronic use can cause neurotoxicity in animals and humans but the long-term consequences are not well understood. The aim of the current study was to investigate the long-term effect of stimulant use on the morphology of the human substantia nigra. We hypothesised that history of illicit stimulant use is associated with an abnormally bright and enlarged substantia nigra (termed ‘hyperechogenicity’) when viewed with transcranial sonography. Substantia nigra morphology was assessed in abstinent stimulant users (n = 36; 31±9 yrs) and in two groups of control subjects: non-drug users (n = 29; 24±5 yrs) and cannabis users (n = 12; 25±7 yrs). Substantia nigra morphology was viewed with transcranial sonography and the area of echogenicity at the anatomical site of the substantia nigra was measured at its greatest extent. The area of substantia nigra echogenicity was significantly larger in the stimulant group (0.273±0.078 cm^2^) than in the control (0.201±0.054 cm^2^; P<0.001) and cannabis (0.202±0.045 cm^2^; P<0.007) groups. 53% of stimulant users exhibited echogenicity that exceeded the 90^th^ percentile for the control group. The results of the current study suggest that individuals with a history of illicit stimulant use exhibit abnormal substantia nigra morphology. Substantia nigra hyperechogenicity is a strong risk factor for developing Parkinson's disease later in life and further research is required to determine if the observed abnormality in stimulant users is associated with a functional deficit of the nigro-striatal system.

## Introduction

Illicit stimulants such as amphetamine, methamphetamine, cocaine, and ecstasy (3,4-methylenedioxymethamphetamine or MDMA) temporarily increase alertness, mood, and euphoria. These effects arise from their acute mechanism of action on the monoamine neurotransmitters dopamine, noradrenaline, and serotonin. There are important differences in the degree to which the different stimulants affect these three neurotransmitters. For example, amphetamine, methamphetamine, and cocaine administration all result in excess accumulation of mainly dopamine [1], [2], [3] whereas ecstasy administration results in accumulation of mainly serotonin and noradrenaline [4]. Animal and in vitro studies show that amphetamine and methamphetamine disrupt synaptic vesicles, inhibit monoamine oxidase [5], [6], and block and/or reverse vesicular monoamine transporters [7], [8]. Furthermore, both amphetamines and cocaine affect dopamine reuptake transporters [7], [8], [9].

Chronic use of illicit stimulants is associated with long-lasting changes in monoamine neurotransmission. Animal studies suggest that the striatum is particularly susceptible to damage from amphetamines. In rats, chronic use of amphetamines is associated with dopamine deficiency and neurotoxicity due to a combination of mechanisms, including mitochondrial dysfunction, oxidative stress, excitotoxicity, and neuroinflammation [10]. In humans, neuroimaging studies also suggest a long-lasting reduction in dopamine reuptake transporter [11] and dopamine (D2) receptor availability [12] in the striatum of abstinent methamphetamine users. Conversely, ecstasy use is associated with long-lasting serotonergic dysfunction (e.g. depletion of 5-HT and decreased SERT density) in rats [13], [14], [15], non-human primates [16], and humans [17], [18], [19] in several brain regions including the basal ganglia (striatum) [20], [21].

The aim of the current study was to investigate the long-term effect of illicit stimulant use on the morphology of the substantia nigra, a midbrain structure with dense projections to the striatum and a high concentration of dopaminergic neurones. The morphology of the substantia nigra is difficult to assess in conscious humans with clinical magnetic resonance imaging, but it can be readily viewed with transcranial sonography [22]. The technique involves placing a low frequency ultrasound transducer at the pre-auricular acoustic bone window (at the orbito-meatal line, above the ear) and measuring the area of echogenicity planimetrically at the anatomical site of the substantia nigra. Measurements are made ipsilateral to the insonating transducer [23].

The sonographic appearance of the substantia nigra is altered in diseases that affect this brain region. For example, the substantia nigra appears abnormally bright and enlarged in 78–90% of Parkinson's disease patients [24], [25], [26], [27] and the abnormality (termed ‘hyperechogenicity’) has a high sensitivity for this condition (positive predictive value: ∼90%) [28], [29]. The mechanisms that contribute to substantia nigra hyperechogenicity are not fully understood but are thought to involve abnormal iron accumulation [30], [31], [32], decreased neuromelanin [32], and activation of microglia [33]. Mutations in genes that are involved in the cellular regulation of iron transport (e.g. ceruloplasmin gene) also appear to be associated with substantia nigra hyperechogenicity [34]. Furthermore, substantia nigra hyperechogenicity is associated with reduced dopaminergic uptake in the striatum of Parkinson's disease patients and healthy adults with substantia nigra hyperechogenicity [35]. Healthy adults with this abnormality (aged over 50 yrs) are also 17 times more likely to develop Parkinson's disease over a 3 yr period [36].

We hypothesise that history of illicit stimulant use is associated with abnormal substantia nigra hyperechogenicity. The hypothesis does not seek to differentiate the effect of specific illicit stimulants on human substantia nigra morphology because individuals tend to use more than one type of stimulant drug during their lifetime.

Evidence that supports our hypothesis comes from the literature on methamphetamine. Methamphetamine treated vervet monkeys exhibit increased iron in the substantia nigra [37] and similarities between the brains of chronic methamphetamine users and Parkinson's disease patients, among whom the incidence of hyperechogenicity is very high [22]. Chronic methamphetamine use and Parkinson's disease affect dopamine release and loss of the ability to recapture dopamine and transport it back inside the cell is a common feature in both conditions [10], [38]. Histopathological studies in humans also suggest increased intracellular inclusions (containing ubiquitin and α-synuclein) in the substantia nigra of Parkinson's disease patients [39] and human methamphetamine users [40]. If our hypothesis is correct, illicit stimulant use may increase the risk of developing a movement disorder later in life given that healthy older adults with this abnormality are 17 times more likely to develop Parkinson's disease over a 3 year period [36].

## Materials and Methods

Substantia nigra morphology was assessed in 79 adults aged 18–50 years. Three groups of subjects were investigated: 37 illicit stimulant users, 12 cannabis users, and 30 non-drug users. The inclusion criteria for the stimulant group were use of amphetamine, methamphetamine, ecstasy and/or cocaine on greater than 3 occasions. Inclusion criteria for the cannabis group were use of cannabis on greater than 5 occasions but no history of stimulant use. The cannabis group acted as a positive control group (i.e. to make sure that changes in substantia nigra echogenicity are not primarily caused by cannabis use) because cannabis use is common among stimulant users. Inclusion criteria for the control group were cannabis use on less than 2 occasions and no other history of illicit drug use (alcohol and tobacco use was permitted). The study was conducted in Adelaide, Australia. Seventy eight volunteers were recruited via community advertisement and one volunteer was recruited from a rehabilitation program (Warinilla Clinic, Drug and Alcohol Services South Australia). All experimental procedures were approved by the Human Research Ethics Committee at the University of South Australia, Royal Adelaide Hospital, and Drug and Alcohol Services South Australia. Experimental procedures were conducted according to The Code of Ethics of the World Medical Association (Declaration of Helsinki) printed in the British Medical Journal (18^th^ July 1964). Written informed consent was obtained prior to participation.

### Subject screening

Subjects underwent a series of screening tests prior to participating in the study. Subjects were asked to complete a brief questionnaire to document age, height, weight, and medical history [41] and provide a urine sample for routine drug screening (PSCupA-6MBAU, US Diagnostics Inc., Huntsville, Alabama, USA). Urine data is missing for 4 subjects (2 control subjects, 1 stimulant subject, and 1 cannabis subject) due to mislabelling of samples, although drug users reported complete drug abstinence for 6 and 13 yrs, respectively. All subjects were then asked to complete an in-house drug history questionnaire to document lifetime and recent use of alcohol, tobacco, and illicit drugs. The questionnaire listed 20 illicit drugs and requested information on other illicit drugs not listed. Items on the questionnaire included age of first use, age of regular use, duration of use, frequency of use (current and lifetime), number of times used in the last year, average dose (current and lifetime), frequency of high dose use, and time since last use defined for each drug and number of drug overdoses and treatment for drug dependency. The final screening test involved a neuropsychological assessment of memory and cognition. Four cognitive domains were assessed. New learning was assessed with Logical Memory I and II [42], executive functioning was assessed with Verbal Trails and Verbal Fluency [43], [44], working memory was assessed with Digit Span backwards [45], and attention was assessed with Digit Span forwards [45]. Performance in each test was compared to published normative data matched for age and years of education. Symptoms of depression (over the past 2 weeks) were also assessed with a 21 item self-report rating scale (Beck Depression Inventory-II) [46].

Exclusion criteria included a) history of neurological damage and/or neurological illness prior to illicit drug use, b) use of antipsychotic medications, c) frequent illicit opioid use (i.e. >2 times per year), and d) positive urine drug test for amphetamine, methamphetamine, MDMA (3,4-methylenedioxymethamphetamine or ‘ecstasy’), cocaine, opioids, and/or benzodiazepines to ensure that neuropsychological testing was unaffected by the acute effects of drug use. Subjects who tested positive for cannabis were allowed to participate if use was greater than 12 hours prior to the experiment. This exemption was due to the metabolite of the main active ingredient of cannabis (tetrahydrocannabinol) remaining in the body for up to 90 days after last use. Subjects were also excluded if poor performance was observed on 3 or more of the cognitive domains tested. Poor performance was defined as greater than 2 standard deviations below the mean of published normative data for digit span [47], verbal fluency [48], and logical memory I and II [49] and performance greater than 2 standard deviations above the mean for verbal trails [50].

### Experimental protocol

The experiment began with subjects completing a modified version of the Edinburgh Handedness Inventory [51] to assess hand dominance. Subjects then underwent transcranial sonography in a supine position. The examination was performed with a Philips iU22 ultrasound system equipped with a 1–5 MHz transducer (s5–1) (Philips Healthcare, Best, The Netherlands). Two iU22 machines were used (machine 1: manufactured Dec 2004 with 2009 software (level 4.1.1.58); machine 2: manufactured June 2004, refurbished November 2011 with 2011 software (level 6.0.2.144)) but the transducer remained constant. The transducer was positioned over the pre-auricular acoustic bone window located above the ear. The penetration depth was set at 14–16 cm and the dynamic range was 50–60 dB. A qualitative rating of the bone window was made (1-excellent, 2-good, 3-poor, 4-very poor) and the area of echogenicity at the anatomical site of the substantia nigra was measured at its greatest extent. Three other parameters were also measured in line with pre-established guidelines [28]: a) minimum internal diameter of the 3^rd^ ventricle, b) area of the red nucleus at its greatest extent, and c) qualitative rating of the raphii nucleus on one (clearest) side (normal, abnormal-interrupted, abnormal-absent). The examination was performed by 1 operator (GT) and measurements were made in the B-mode setting.

In 21 subjects (17 controls, 3 cannabis, 1 stimulant), the ultrasound procedure was repeated immediately by a second operator (CN) for quality control. Inter-rater variability and reproducibility were calculated. A subset of still images and movie files were also forwarded to a third rater (DB) for an expert and blinded opinion.

### Data analysis

Group data are presented as the mean ± standard deviation (SD). Between-group comparison of subject characteristics (age, height, weight, years of education), neuropsychological parameters, and ultrasound parameters was made with one-way analysis of variance (ANOVA). Non-parametric data were transformed to ranks and ANOVA on ranks were performed. Post-hoc discrimination between means was made with Student-Newman Keuls procedure. Unpaired Student's t-test was used to compare cannabis parameters between the stimulant and cannabis groups. Paired Student's t-test was used to compare lifetime use of ecstasy and amphetamine-like stimulants within the stimulant group. Spearman Rank Order correlation was used to investigate the relationship between area of substantia nigra echogenicity (largest side) and drug-use and neuropsychological parameters (SigmaPlot 11.0, Systat Software Inc, Chicago, USA). Inter-rater reliability was assessed with Cronbach's alpha and Spearmann Rank Order correlation. Inter-rater reproducibility was assessed with the intraclass correlation coefficient (IBM SPSS Statistics Version 20, IBM, Armonk, New York, USA). Comparison of measurements obtained on machine 1 and 2 in the control group was made with unpaired Student's t-test (SigmaPlot 11.0, Systat Software Inc, Chicago, USA). Significance was set at P<0.05.

## Results

### Subject characteristics

Two subjects were excluded due to insufficient bone window for transcranial sonography (1 control and 1 stimulant user). The characteristics of the remaining 77 subjects are presented in Table 1. There was a significant difference between the groups regarding age (F_2,74_ = 8.007, P<0.001) but not weight or height. The average age of subjects in the stimulant group was ∼6.5 yrs older than subjects in the control (P = 0.001) and cannabis groups (P = 0.009). There was also a significant main effect of group on years of education (F_2,74_ = 3.268, P = 0.044) and a trend for a main effect of group on symptoms of depression (i.e. BDI-II score; F_2,73_ = 2.743, P = 0.071). Subjects in the stimulant group had undertaken ∼1 less year of education compared to the control group (P = 0.041) and subjects in the stimulant and cannabis groups tended to have more symptoms of depression. Seven subjects in the stimulant group and 3 subjects in the cannabis group had received a formal diagnosis of depression (4 were currently medicated). The date of diagnosis occurred between 2 and 27 yrs after commencement of illicit drug use. All subjects exhibited normal neuropsychological performance and the groups did not significantly differ on the Logical Memory I and II, Verbal Fluency, and Digit Span forwards and backwards tests. However, subjects in the control group exhibited poorer performance on the Verbal Trails test (33±10 s) than subjects in the stimulant (29±13 s) and cannabis (25±8 s) groups (P<0.046).

**Table 1:** Subject characteristics for the control, stimulant, and cannabis groups.

	Control (n = 29)	Stimulant (n = 36)	Cannabis (n = 12)
Age (yrs)	24±5	31±9 * §	25±7
Gender	11 M, 18 F	21 M, 15 F	6 M, 6 F
Weight (kg)	69±22	74±16	75±21
Height (cm)	161±37	165±41	170±11
Handedness	23 right, 6 left	32 right, 4 left	10 right, 2 left
Education (yrs)	16±2	15±3 *	15±2
BDI-II score	6±6	10±8	12±10
Depression diagnosis	0	7	2
Head injuries	0	10	1
Drug overdose	0	4	0
Lifetime alcohol (total drinks)	479±620	12,384±15,661 * §	2,244±2,159 *
Lifetime tobacco (total cigarettes)	171±915	64,096±101,413 * §	11,011±23,314 *

Data are mean±standard deviation.

* Significantly different from control group (P<0.05).

§ Significant difference between stimulant group and cannabis group (P<0.05).

### Drug history

Use of alcohol and tobacco was significantly different between groups (alcohol: F_2,69_ = 46.799; P<0.001, tobacco: F_2,74_ = 49.576; P<0.001; Table 1). Lifetime use of alcohol (estimated total drinks) and tobacco (estimated total cigarettes) was greatest in the stimulant group and least in the control group (P<0.007).

Table 2 shows the percentage of subjects within each group that had used various classes of illicit drugs. Ecstasy was the most commonly used stimulant followed by methamphetamine, cocaine, and recreational use of pharmaceutical stimulants. Poly-drug use was common in the stimulant group and less common in the cannabis group. All subjects in the stimulant group had used cannabis and the majority of subjects had used hallucinogens (primarily lysergic acid diethylamide or ‘LSD’) and inhalants (primarily nitrous oxide). Illicit use of sedatives and opiates was uncommon and total lifetime use of these drugs was low (sedatives: 25±70 occasions; opiates: 5±8 occasions).

**Table 2:** Classes of illicit drugs consumed in the stimulant and cannabis groups.

	Stimulant group	Cannabis group
Stimulants	100%	0%
Ecstasy	94%	0%
Methamphetamine	81%	0%
Cocaine	56%	0%
Pharmaceutical	25%	0%
Cannabis	100%	100%
Hallucinogens	86%	17%
Inhalants	67%	25%
Sedatives	39%	8%
Opiates	36%	0%
Overdoses	4	0

Data are percentage of subjects that have consumed that class of illicit drug in their lifetime. The term ‘hallucinogen’ describes LSD (lysergic acid diethylamide), LSA (d-lysergic acid amide), ‘magic’ mushrooms, DOI (2,5-dimethoxy-4-iodoamphetamine), salvia divinorum, ayahuasca, DMT, ketamine, and/or mescaline. The term ‘opiate’ describes heroin, methadone, opium, poppy tea, and recreational use of codeine, oxycodeine, hydrocodeine, and/or morphine. The term ‘inhalant’ describes amyl nitrate, nitrous oxide, and/or glue. The term ‘sedative’ describes GHB/Fantasy, methaqualome, chelidonium majus, and recreational use of benzodiazepine, antidepressants, and antihistamine.

Table 3 shows single subject and group data for lifetime use of ecstasy, amphetamine-like stimulants, and cannabis in the stimulant group. Lifetime use of amphetamine-like stimulants was significantly greater than ecstasy (P = 0.004) and lifetime use of cannabis tended to be greater in the stimulant group than in the cannabis group (305±549 occasions; P = 0.09). The average duration of stimulant use was 8.1±6.8 yrs (range: 3 days-27 yrs) and the average duration of abstinence from stimulants was 2.0±3.6 yrs (range: 3 days-15 yrs). The average duration of abstinence from cannabis was 0.5±1.3 yrs (range: 1 day-6 yrs) and there was a tendency for a longer duration of cannabis abstinence in the cannabis group (1.8±3.8 yrs, range: 1 day-13 yrs; P = 0.07).

**Table 3:** Summary of lifetime use of stimulants and cannabis in the stimulant group.

Subject	Total stimulants	Amphetamines	Ecstasy	Cannabis
1	3029	3029	0	5475
2	2967	2651	317	5840
3	2241	2072	169	28
4	2059	1851	208	4745
5	1576	1560	16	15
6	1396	1034	362	8212
7	875	719	156	228
8	833	832	1	13
9	670	520	150	1140
10	387	327	60	54
11	367	211	156	4380
12	332	228	104	1251
13	247	244	3	7365
14	234	231	4	360
15	209	208	1	6570
16	204	164	40	33945
17	139	14	125	1104
18	86	13	73	128
19	79	35	44	11315
20	57	5	52	4380
21	36	10	26	474
22	32	12	20	832
23	27	26	1	270
24	19	8	11	6
25	19	1	18	15
26	16	1	15	20
27	14	9	5	10741
28	13	1	12	2555
29	12	3	9	72
30	7	7	0	4384
31	7	1	6	183
32	6	1	5	60
33	6	4	2	9855
34	6	0	6	260
35	3	0	3	104
36	3	0	3	15
Mean	506	486	64	3511
(SD)	(845)	(820)	(92)	(6256)

Single subject and mean data are presented (number of times used). The term ‘amphetamine’ describes amphetamine and amphetamine-like drugs such methamphetamine, cocaine, dexamphetamine, Ritalin®, and khat (1 subject). The term ‘ecstasy’ describes ecstasy, MDA (3,4-methylenedioxyamphetamine, 2 subjects), and MCAT (mephedrone, 1 subject).

### Transcranial ultrasound

The maximum subjective rating of the bone window was calculated for each subject and the average was 1.6±0.8 (i.e. good to excellent; median = 1 excellent). The diameter of the 3^rd^ ventricle was normal in all subjects (maximum diameter: 4.94 mm) and the average diameter (right, left) did not significantly differ between groups (control: 1.51±0.08 mm, stimulant: 1.44±0.07 mm; cannabis: 1.04±0.03 mm).

**Figure 1:**
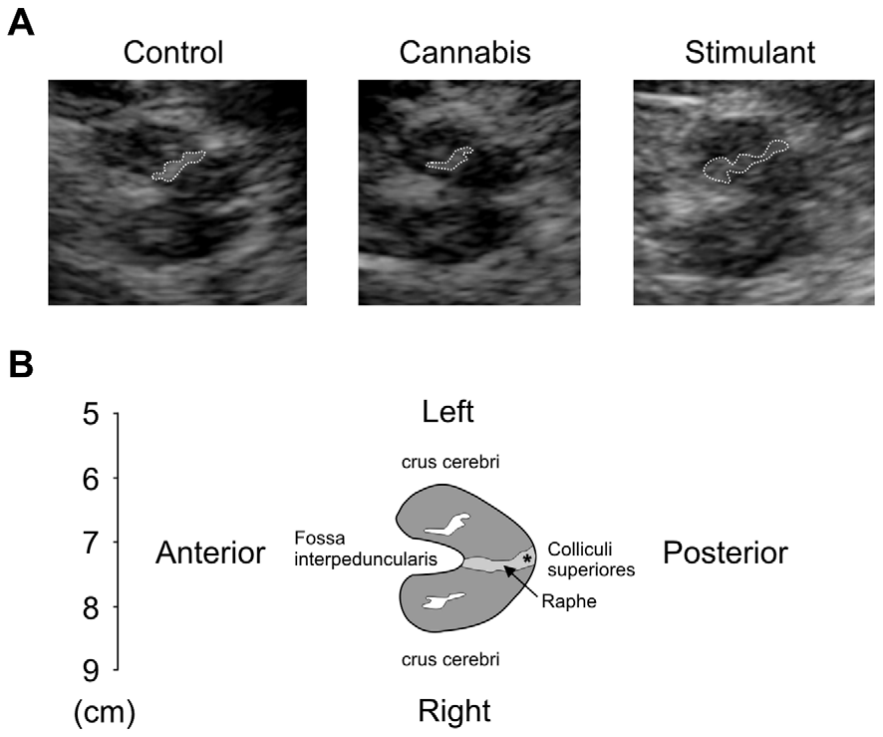
Single subject data showing echomorphology of the mesencephalic brainstem. A) Images from 1 control subject, 1 cannabis subject, and 1 stimulant subject. The substantia nigra ipsilateral to the probe (the side at which the planimetric measurement is done) is encircled with a dotted line. B) Schematic drawing of the mesencephalic brainstem. * aqueduct. Raphe, echogenicity of midline structures.

Figure 1A shows single subject images of the area of substantia nigra echogenicity in 1 control subject, 1 cannabis subject, and 1 stimulant subject. For a given side (right), the average area of substantia nigra echogenicity was 0.163±0.044 cm^2^ for operator 1 and 0.166±0.051 cm^2^ for operator 2. The area of substantia nigra echogenicity exhibited acceptable inter-rater reliability (Cronbach's alpha = 0.720; Spearman rank order correlation: r = 0.591, P = 0.005) with moderate to strong reproducibility (intraclass correlation coefficient; single measures = 0.577; average measures = 0.732). There was no significant difference between measurements obtained on machine 1 and 2 in the control group. Single subject data suggested that the area of substantia nigra echogenicity was greater in stimulant subjects than in control and cannabis subjects.

**Figure 2:**
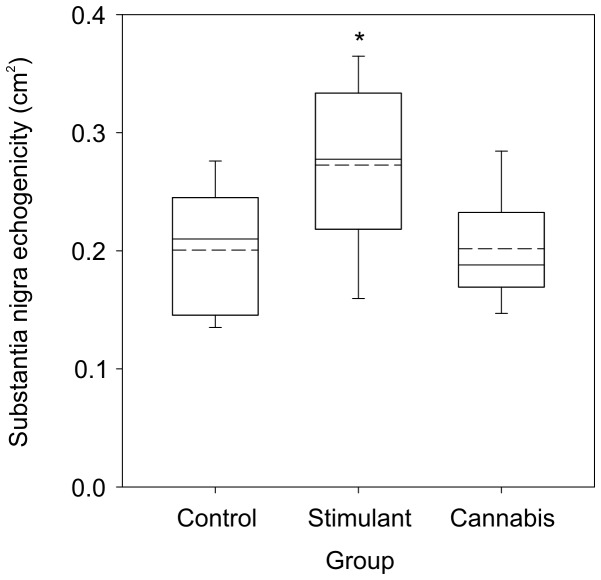
Group data showing the area of substantia nigra echogenicity. Data represent the largest area across the right and left side. Data for the control, stimulant, and cannabis groups are shown. The boundary of each box indicates the 25^th^ and 75^th^ percentile. The solid and dashed lines within each box indicate the median and mean values, respectively. * significant difference from control and cannabis groups (P<0.007).

Figure 2 shows group data for the area of substantia nigra echogenicity. In the control group, the average area of substantia nigra echogenicity was 0.181±0.055 cm^2^ on the right side (median: 0.175) and 0.176±0.057 cm^2^ on the left side (median: 0.180). The area of substantia nigra echogenicity significantly differed between groups. The significant difference occurred for both the average area (right, left; F_2,74_ = 9.590; P<0.001) and largest area (right or left, F_2,74_ = 10.206; P<0.001). Post hoc analysis revealed that area of echogenicity was significantly larger in the stimulant group than in the control (P<0.001) and cannabis (P<0.007) groups. In the stimulant group, 53% of subjects exhibited echogenicity that exceeded the 90^th^ percentile for the control group (i.e. >0.258 cm^2^). There was no significant difference between the area of substantia nigra echogenicity in the control and cannabis groups.

In the stimulant group, there was a significant positive correlation between the largest area of substantia nigra echogenicity (right or left) and lifetime use of hallucinogens (P = 0.002, correlation coefficient = 0.536). There was also a trend for a positive correlation between substantia nigra echogenicity and lifetime use of tobacco (P = 0.051, correlation coefficient = 0.328) and inhalants (P = 0.067, correlation coefficient = 0.379). There was no significant correlation between the area of substantia nigra echogenicity and other drug use parameters or neuropsychological parameters. In the cannabis group, there was no correlation between the area of substantia nigra echogenicity and lifetime tobacco use. The relationship between area of substantia nigra echogenicity and lifetime use of hallucinogens (n = 2) and inhalants (n = 3) could not be investigated due to small numbers.

The red nucleus was clearly delineated bilaterally in 71 subjects and unilaterally in 6 subjects. The average area (right, left) and largest area (right or left) of the red nucleus did not significantly differ between groups (average area; control: 0.055±0.014 cm^2^, stimulant: 0.054±0.015 cm^2^, cannabis: 0.049±0.010 cm^2^). The raphe was rated abnormal-interrupted in 7%, 17%, and 25% of subjects in the control, cannabis, and stimulant groups, respectively. The raphe was rated normal in all other subjects.

## Discussion

We used transcranial sonography to investigate substantia nigra morphology in healthy adults with a history of illicit stimulant use. The results of our study show for the first time that individuals with a history of primarily methamphetamine and ecstasy use exhibit abnormal substantia nigra morphology. The abnormality (termed hyperechogenicity) is long lasting and does not appear to be associated with concurrent cannabis use.

The area of substantia nigra echogenicity was significantly larger in individuals with a history of illicit stimulant use than in non-drug users and cannabis users. The hyperechogenicity observed in stimulant users (0.273±0.078 cm^2^) was comparable to older adults with clinical Parkinson's disease (0.275–0.34 cm^2^) [52], [53]. Identifying the underlying mechanism for the hyperechogenicity is difficult in human drug users. We can conclude that the abnormality is not associated with the acute mechanism of action of stimulants because the average duration of abstinence was 2±3 years and subjects had a negative urine screen for stimulants, opiates, and benzodiazepines. The abnormality is also not associated with changes in memory, cognition, and gross brain volume because all subjects passed neuropsychological screening and all subjects exhibited a normal ventricular system. The abnormality is also unlikely due to drug overdose because only 4 subjects reported experiencing such an event. However, beyond that one can only speculate due to methodological limitations associated with all studies on illegal stimulant use in humans. For example, no two people exhibit the same drug use pattern, lifestyle, or environment and there are challenges associated with self-reporting of lifetime drug use and difficulty in obtaining accurate information on the dose and composition of the substances used. Table 2 highlights another significant challenge, poly-drug use. In the current study, 94% of subjects in the stimulant group had used ecstasy, 81% had used methamphetamine, and 56% had used cocaine. Poly-stimulant use is well documented in the literature and is clearly evident in national drug surveys [54]. Cannabis use is also very common amongst stimulant users, with over 70% of stimulant users reporting concurrent cannabis use [54]. Furthermore, stimulant users consume more alcohol [55] and tobacco [56] than non-drug users. Thus, in humans, it is difficult to ascribe an observed abnormality to a specific drug but changes can be ascribed to a class of drug (e.g. stimulants) with careful experimental design and control measures.

It is mechanistically plausible that use of each of the three illicit stimulants, methamphetamine, cocaine, and ecstasy, contributed to the abnormal substantia nigra morphology in the stimulant group. There are a number of lines of evidence to support this view. In particular, methamphetamine exposure has been directly linked with changes in the substantia nigra. Adult vervet monkeys treated with 2 doses of methamphetamine (2 mg/kg) exhibit a 1–2 fold increase in the intensity of iron staining in the substantia nigra at 1-month post-drug administration and a 2.5 fold increase in iron staining intensity at 1.5 yrs post-drug administration [37]. Additionally, pre-synaptic dopaminergic dysfunction (i.e. reduced [^18^F]-dopa activity) is present in vervet monkey striatum 24 weeks after a 10-day period of amphetamine administration (4–18 mg/kg/day) [57] and pre-synaptic dopaminergic dysfunction is also present in the striatum of healthy young adult humans with substantia nigra hyperechogenicity [31], [58]. In cocaine dependent individuals, increased activation of microglia is present in the substantia nigra at post-mortem [59] and increased activation of microglia is associated with substantia nigra hyperechogenicity in healthy adults [33]. Finally, amphetamine, methamphetamine, and cocaine damage dopaminergic nerve terminals and chronic use of amphetamines is associated with long-lasting dopaminergic dysfunction [3], [10].

Concurrent use of stimulants and tobacco, hallucinogens, and inhalants could also have contributed to the abnormal substantia nigra morphology. In the stimulant group, there was a positive correlation between the area of substantia nigra echogenicity and lifetime use of hallucinogens and a trend for a positive correlation with lifetime use of tobacco and inhalants. The most commonly used hallucinogens were LSD and ‘magic’ mushrooms (likely *Psilocybe* species). The psychoactive compound in LSD and *Psilocybe* exhibits a chemical structure that resembles serotonin. These drugs are considered to have a low level of toxicity but *Psilocybe* can be mistaken for other varieties that have poisonous, and sometimes lethal, effects. In regards to tobacco, cigarettes contain chemicals that are monoamine oxidase inhibitors [60]. Concurrent use of tobacco and amphetamines may facilitate the effect of amphetamines on nerve terminals by impairing degradation of monoamine neurotransmitters. Use of cannabis and opiates is unlikely to have had a strong effect given that illicit opiate use was minimal in the current cohort and substantia nigra morphology was normal in cannabis users.

The results of the current study cause one to speculate about the potential association between chemical exposure and substantia nigra hyperechogenicity. One study that supports a link between chemical exposure and substantia nigra echogencity involved administration of 1-methyl-4-phenyl-1,2,3,6-tetrahydropyridine (MPTP) to Rhesus monkeys [61]. However, the type and/or concentration of exposure is likely to be important given that substantia nigra echogenicity was not corrected to lifetime stimulant use (number of occasions) in the current study and substantia nigra echogenicity in older patients with Parkinson's disease does not significantly differ with exposure to pesticides, herbicides, paint, solvents, and heavy metals [24]. However, the latter substances cannot be readily linked mechanistically with changes in dopamine neurons induced by stimulants.

Other factors that could have contributed to abnormal substantia nigra hyperechogenicity in stimulant users are age and increased symptoms of depression. Individuals in the stimulant group were ∼7 yrs older than subjects in the control and cannabis groups and drug users tended to exhibit more symptoms of depression than non-drug users. However, the contribution of these factors to substantia nigra hyperechogenicity in the stimulant group is likely to be minimal. The area of substantia nigra echogenicity does not vary between 20 to 50 yrs of age [58] and cannabis users exhibited normal substantia nigra morphology despite a tendency for a higher score on the Beck Depression Inventory-II. Substantia nigra hyperechogenicity could also be associated with personality traits in stimulant users. Individuals in the stimulant group had received 1 year less of education and substance-dependence is associated with impulsive behavior [62]. However, the contribution of personality traits to the result is likely to be small given that personality traits do not correlate with substantia nigra echogenicity in patients with Parkinson's disease [24]. Furthermore, cannabis users exhibited normal substantia nigra morphology and lifetime initiation of cannabis use typically precedes stimulant use. In the current cohort of stimulant users, cannabis use commenced at age 17±3 yrs whereas stimulant use commenced at age 20±5 yrs (paired t-test, P<0.001). Substantia nigra morphology was also normal in 3 cannabis subjects with heavy cannabis use (>4 days per week) and signs of addictive behaviour (i.e. daily use).

### Ultrasound measurement validity

In the control group, the area of substantia nigra echogenicity (average: 0.176–0.181 cm^2^; median: 0.175–0.180 cm^2^) was higher than that reported previously (median: 0.11–0.14 cm^2^) [24], [34], [58], [63]. However, the 90^th^ percentile (0.258 cm^2^) was similar to a previous study involving 301 healthy individuals (0.25 cm^2^) [58] and areas ranging from 0.28–0.35 cm^2^ have been previously reported in healthy young adults [31]. The higher area of echogenicity in the current study is likely due to differences in the ultrasound manufacturer, transducer properties (1–5 MHz versus 2.5 MHz), greater propensity for the ultrasound beam to penetrate bone (97.5% versus 77–92%) [24], [52], [53], [58], and improvements in ultrasound resolution over time. Such factors did not contribute to the between group difference observed in the current study because all subjects were tested with a Philips iU22 system and s5–1 transducer.

The between group difference in substantia nigra echogenicity is also unlikely due to the ultrasound operator. All subjects were tested by one operator and the measurements collected by this operator were consistent with those collected immediately after by a second operator. The reliability and reproducibility statistics were comparable to those published previously [63], [64] and a subset of images was viewed by a third person for confirmation of image quality. However, a limitation of the current study is that the operator was not blinded to the individual's drug history. Appropriate blinding was not possible due to limited resources and personnel. Future studies will need a double-blind design to confirm the current findings.

## Conclusions

The results of the current study suggest that some individuals with a history of illicit stimulant use exhibit abnormal substantia nigra morphology. Substantia nigra hyperechogenicity is a strong risk factor for developing Parkinson's disease later in life [36] and our result supports recent epidemiological data suggesting that methamphetamine use is associated with increased risk (hazard ratio = 2.65) of developing Parkinson's disease [65]. Further research is required to determine if the observed abnormality in stimulant users is associated with subtle movement dysfunction.
